# Intravascular Laser Blood Irradiation (ILIB) Enhances Antioxidant Activity and Energy Metabolism in Aging Ovaries

**DOI:** 10.3390/jpm14060551

**Published:** 2024-05-22

**Authors:** Li-Te Lin, Chia-Jung Li, Chyi-Uei Chern, Pei-Hsuan Lin, Po-Wen Lin, Yu-Chen Chen, Hsiao-Wen Tsai, Kuan-Hao Tsui

**Affiliations:** 1 Department of Obstetrics and Gynaecology, Kaohsiung Veterans General Hospital, Kaohsiung 813, Taiwan; 2 Department of Nursing, Shu-Zen Junior College of Medicine and Management, Kaohsiung 821, Taiwan; 3 Institute of Biopharmaceutical Sciences, National Sun Yat-sen University, Kaohsiung 804, Taiwan; 4 Department of Obstetrics and Gynaecology, National Yang-Ming University School of Medicine, Taipei 112, Taiwan; 5 Department of Obstetrics and Gynecology, Taipei Veterans General Hospital, Taipei 112, Taiwan; 6 Department of Medicine, Tri-Service General Hospital, National Defense Medical Center, Taipei 114, Taiwan

**Keywords:** ILIB, ovarian aging, oxeiptosis, energy metabolism

## Abstract

Background: Ovarian aging is characterized by the accumulation of free radicals, leading to tissue damage and affecting reproductive health. Intravascular laser irradiation of blood (ILIB, using a low-energy He-Ne laser) is known for its efficacy in treating vascular-related diseases by reducing free radicals and inflammation. However, its impact on ovarian aging remains unexplored. This study aimed to investigate the effects of ILIB on oxidative stress and energy metabolism in aging ovaries. Methods: Genetic analysis was conducted on 75 infertile patients with aging ovaries, divided into ILIB-treated and control (CTRL) groups. Patients underwent two courses of laser treatment, and clinical parameters were evaluated. Cumulus cells were collected for the genetic analysis of oxeiptosis, glycolysis, and the tricarboxylic acid (TCA) cycle. Results: The analysis of gene expression patterns revealed intriguing findings in ILIB-treated patients compared to the untreated group. Notably, ILIB treatment resulted in significant upregulation of oxeiptosis-related genes AIFM1 and NRF2, suggesting a potential protective effect against oxidative stress-induced cell death. Furthermore, ILIB treatment led to a downregulation of glycolysis-associated gene hexokinase 2 (HK2), indicating a shift away from anaerobic metabolism, along with an increase in PDHA levels, indicative of enhanced mitochondrial function. Consistent with these changes, ILIB-treated patients exhibited elevated expression of the key TCA cycle genes citrate synthase (CS), succinate dehydrogenase complex subunit A (SDHA), and fumarate hydratase (FH), signifying improved energy metabolism. Conclusion: The findings from this study underscore the potential of ILIB as a therapeutic strategy for mitigating ovarian aging. By targeting oxidative stress and enhancing energy metabolism, ILIB holds promise for preserving ovarian function and reproductive health in aging individuals. Further research is warranted to elucidate the underlying mechanisms and optimize the application of ILIB in clinical settings, with the ultimate goal of improving fertility outcomes in women experiencing age-related ovarian decline.

## 1. Introduction

Ovarian aging represents a natural physiological phenomenon characterized by a gradual deterioration in reproductive function, particularly evident in women aged 35 and above [1,2]. This process presents itself as diminished ovarian reserve, compromised quality of oocytes, and an increased occurrence of chromosomal abnormalities. Together, these factors contribute to reduced fertility and heightened vulnerability to infertility, as well as complications during pregnancy [2,3,4]. Various factors contribute to this age-associated decline, including diminished mitochondrial function and antioxidant capacity within both the oocyte and surrounding cumulus granulosa cells [5,6,7,8]. Mitochondria play a pivotal role in cellular energy production through oxidative phosphorylation, serving as vital regulators of energy metabolism, metabolic homeostasis, and oxidative stress management, thus exerting significant influence over reproductive aging processes. Within germ cells, mitochondria fulfill indispensable roles in supporting energy-intensive processes such as oocyte maturation, fertilization, and subsequent embryonic development [9,10,11,12,13].

In the analysis of real-world data, it became evident that individuals categorized as younger or older expected adverse responders exhibited notably lower cumulative delivery rates compared to those classified as normal responders (29.4% vs. 12.5% vs. 50.6%, respectively) [14]. The risk of encountering poor ovarian response (POR) during subsequent in vitro fertilization (IVF) cycles was approximately 11.5%, with a substantially elevated risk observed among women with low ovarian reserve, experiencing POR again in 57% of cases within one year following initial IVF attempts [15]. Furthermore, a significant proportion of cycles (13.3%) yielded no high-quality embryos, underlining the prevalence of scenarios characterized by POR and poor embryo quality [16]. These clinical presentations underscore the complex nature of ovarian aging, where a woman’s chronological age serves as a fundamental predictor, yet premature ovarian aging can also manifest, leading to compromised oocyte quality and subsequent poor embryo development.

The etiology of ovarian aging involves multifactorial processes, with oxidative stress emerging as a pivotal contributor [17]. Oxidative stress, characterized by an imbalance between prooxidants and antioxidants in favor of the former, can exert deleterious effects on ovarian tissue and function, ultimately leading to diminished ovarian reserve and compromised oocyte quality [18]. To mitigate the clinical manifestations associated with ovarian aging, antioxidant interventions have been explored. These interventions encompass substances such as vitamins C and E, coenzyme Q10, and melatonin, which aim to counteract the harmful effects of oxidative stress and restore cellular homeostasis [19]. However, the efficacy of these interventions, particularly in individuals with poor ovarian response, has been limited, necessitating the exploration of novel therapeutic modalities.

Intravascular laser irradiation of blood (ILIB) has emerged as a promising therapeutic approach for mitigating oxidative stress and inflammation, as well as promoting tissue regeneration and repair [20]. ILIB entailed exposing circulating blood to low-intensity laser irradiation, a process known to stimulate mitochondrial activity, augment cellular metabolism, and regulate inflammatory responses. This treatment modality involved directing a low-intensity laser beam onto the bloodstream, where it interacted with blood cells, potentially influencing various physiological processes [21]. These effects are attributed to the activation of cellular signaling pathways, including the upregulation of antioxidant enzymes and the modulation of proinflammatory cytokine production [22]. Given the promising anti-aging, antioxidant, and anti-inflammatory properties of ILIB, our study aims to delve into its potential therapeutic effects in individuals with poor ovarian response and concurrent poor embryo quality.

In our study, we enrolled 75 infertile patients exhibiting indications of ovarian aging who underwent ILIB treatment, which involves the insertion of a laser fiber into a vein via a catheter. Each treatment session spanned 60 min, with two sessions administered one week apart to optimize therapeutic efficacy. Our comprehensive assessment protocol includes the evaluation of changes in proinflammatory cytokine profiles, hormonal dynamics, and anti-Müllerian hormone (AMH) levels before and after ILIB treatment. Additionally, we will meticulously analyze the quantity and quality of retrieved oocytes, fertilization rates, embryo morphology, and ultimately, pregnancy outcomes.

Through this comprehensive approach, we aim to elucidate the potential of ILIB as a therapeutic intervention to ameliorate pregnancy outcomes in individuals grappling with poor ovarian response. By promoting mitochondrial function regeneration, enhancing pro-oxidant/antioxidant balance, and modulating inflammatory responses, ILIB may offer a novel therapeutic strategy for individuals struggling with poor ovarian response and poor embryo quality. Furthermore, our study seeks to advance our understanding of the underlying mechanisms involved in ovarian aging and identify potential targets for future therapeutic interventions aimed at optimizing fertility outcomes in this patient population.

## 2. Materials and Methods

### 2.1. Patients and Design

This prospective study enrolled patients with a history of at least two previous IVF failures following the transfer of good-quality embryos at the Reproductive Center of the Kaohsiung Veterans General Hospital in Taiwan between June 2023 to June 2024. Ethical approval was obtained from the Institutional Review Board at the Kaohsiung Veterans General Hospital (VGHKS23-CT3-20), and all participants provided written informed consent. The authors declare no conflicts of interest related to any products used in this study. Participants with recurrent implantation failure who underwent an IVF/embryo transfer cycle utilizing cryopreserved embryos and failed to achieve pregnancy after at least two embryo transfers, each involving a minimum of four top-quality embryos, were prospectively recruited. Inclusion criteria encompassed (i) age between 31 and 44 years; and (ii) a body mass index (BMI) of ≤29 kg/m^2^. Exclusion criteria included (i) severe endometriosis; (ii) uterine anomaly; (iii) adenomyosis; and (iv) the presence of systemic diseases such as autoimmune disorders.

### 2.2. Treatment Protocol

**Figure 1:**
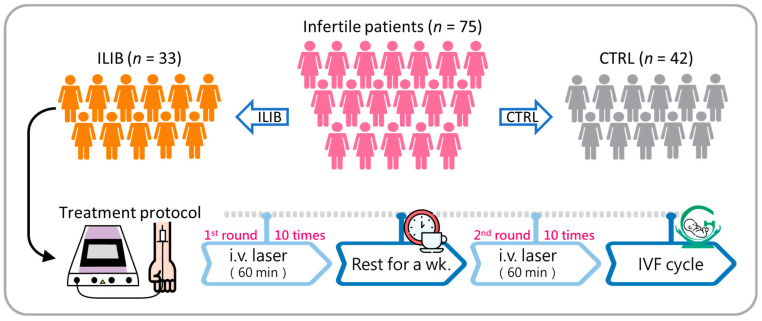
The flowchart provides a visual depiction of the systematic methodology employed throughout the research process to ensure the meticulous selection of relevant studies for inclusion in the analysis.

Patients in the laser group underwent venous blood irradiation using a helium–neon laser (JC-ILIB-800, Jin-Cheng Medical Company Limited, Taipei, Taiwan). Each patient underwent a comprehensive laser treatment protocol comprising a total of 20 sessions administered over a specified period (Figure 1). The treatment protocol commenced with 10 laser sessions, followed by a one-week intermission for recuperation and assessment. Subsequently, patients underwent an additional 10 laser sessions to complete the treatment cycle. Throughout each session, a laser emitting wavelengths of 632.8 nm was employed. The laser output power was meticulously adjusted during the treatment process to optimize therapeutic outcomes while prioritizing patient safety. Initially, the laser was set at a conservative output power of 3.8 mW for the first session, progressively increasing to 4.1 mW in the second session, 4.4 mW in the third session, and 4.7 mW in the fourth session. Subsequently, from the fifth session to the twentieth session, a constant output power of 5.0 mW was maintained. This tailored laser treatment regimen was devised to offer precise therapeutic intervention while mitigating potential adverse effects. Each treatment session was closely monitored to ensure accurate administration of therapeutic laser energy, maximizing its beneficial effects for patients undergoing treatment for their specific condition.

### 2.3. Collection of Patients’ Cumulus Cells

After oocyte retrieval, the patient’s cumulus–oocyte complexes were collected and subsequently washed before being placed in IVF medium under paraffin oil. The cumulus cells were treated with 40 IU/mL hyaluronidase (SynVitro™ Hyadase, Origo, Knardrupvej, Denmark) for 3 min and then washed with phosphate buffered saline (PBS). Following oocyte removal, the cumulus cells were mechanically dissociated and washed again. The resulting cumulus cell pellet was resuspended in Histopaque 1077 (Sigma-Aldrich, Waltham, MA, USA), supplemented with 10% fetal bovine serum (FBS) (Gibco, Thermo Fisher Scientific, Waltham, MA, USA), 5 mg/L insulin, 5 mg/L transferrin, 5 μg/L sodium selenite (ITS, Sigma), and 1.25 μM Androstenedione (4-androstene-3, 17-dione, Sigma). The cumulus cells were then plated in a 4-well plate at a concentration of 2 × 104 viable cells/well and cultured at 37.5 °C in a humidified incubator with 5% CO_2_ for up to 24 h for further experimentation.

### 2.4. Ribonucleic Acid (RNA) Extraction and Real-Time Polymerase Chain Reaction (PCR)

Total RNA extraction followed a standard protocol using QIAzol reagent (QIAGEN). Real-time quantitative PCR (qRT-PCR) analysis was performed using an ABI Prism 7700 Sequence Detection System (Perkin-Elmer Applied Biosystems, Norwalk, CT, USA) and the SYBR Green PCR Core Reagents kit (Perkin-Elmer Applied Biosystems, Norwalk, CT, USA). Duplicate experiments were conducted for each data point, and samples exhibiting a coefficient of variation for Ct value > 1% underwent retesting. Messenger RNA (mRNA) expression levels were assessed using SYBR Green-based qRT-PCR assays on StepOne instrumentation manufactured by Applied Biosystems (Perkin-Elmer Applied Biosystems, Norwalk, CT, USA), known for its accuracy and reproducibility in gene expression analysis. RNU6-1 was employed as a reference gene to ensure the precise normalization of expression data in the qRT-PCR analysis.

### 2.5. Statistical Analysis

The results derived from this study underwent rigorous analysis, encompassing at least three separate experimental runs, to ensure reliability and consistency. All data were presented as the mean value accompanied by the standard deviation to provide a comprehensive overview of the variability within the dataset. Statistical analyses were performed utilizing a two-tailed paired Student’s *t*-test, a commonly employed method to assess the significance of observed differences between experimental conditions. A threshold of *p* < 0.05 was established to determine statistical significance, thereby ensuring robustness and reliability in the interpretation of the experimental outcomes.

## 3. Results

### 3.1. Demographic and Clinical Characteristics of Infertile Patients

A cohort of 75 individuals experiencing infertility was carefully selected from the IVF Center at Kaohsiung Veterans General Hospital to participate in this study. These participants were divided into two distinct groups: the control group (CTRL) and the ILIB group. Detailed demographic and clinical data were meticulously collected and analyzed for each individual. Table 1 and Table 2 provide a comprehensive overview of baseline characteristics, including key variables such as age, BMI, duration of infertility, history of previous IVF failures, and the etiology of infertility. Additionally, various clinical parameters were recorded and analyzed, such as basal levels of follicle-stimulating hormone (FSH), estradiol (E2), and luteinizing hormone (LH); stimulation duration; number of oocytes retrieved; number of metaphase II oocytes; maturation rate; number of fertilized oocytes; fertilization rate; number of Day 3 embryos; and number of top-quality Day 3 embryos.

Furthermore, these tables also include information regarding any underlying medical conditions, medication usage, and lifestyle factors that might influence the study outcomes. This thorough documentation enables a comprehensive understanding of the patient cohort and facilitates meaningful comparisons between the aging and aging/ILIB groups. By incorporating a diverse array of demographic and clinical variables, this detailed profiling allows researchers to explore potential associations between these factors and the response to ILIB therapy. Additionally, it offers valuable insights into the heterogeneity of the patient population, highlighting individual variations that may impact treatment responses and outcomes.

### 3.2. ILIB Treatment Improved Oxeiptosis of Aging Ovarian Cells

**Figure 2:**
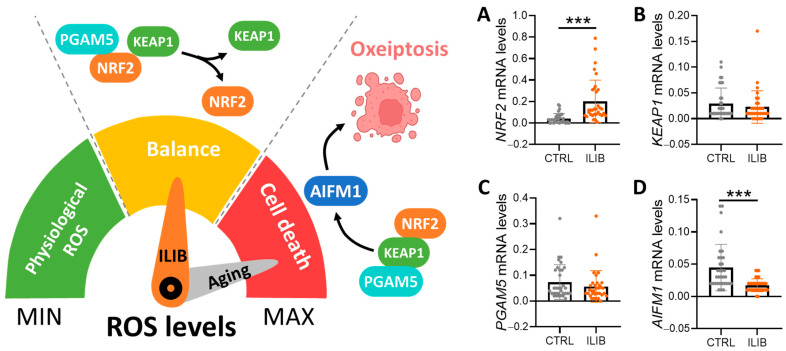
ILIB regulated the oxeiptosis in human cumulus cells. Evaluation of the expression of ILIB altering metabolic pathways and gene levels involved in NRF2 (**A**), KEAP1 (**B**), PGAM5 (**C**) and AIFM1 (**D**) by qPCR analysis. *** *p* < 0.001.

Oxeiptosis has recently been recognized as a distinct form of programmed cell death, characterized by non-inflammatory mechanisms independent of caspase activity. This process involves the detection of reactive oxygen species (ROS) by Kelch-like ECH-associated protein 1 (KEAP1), leading to the activation of apoptosis-inducing factor 1 (AIFM1) and phosphoglycerate mutase 5 (PGAM5), ultimately triggering apoptosis-like cell death. While ROS are typically cleared rapidly under normal conditions, their excessive accumulation can induce various cell death pathways, including apoptosis and ferroptosis. Nuclear factor erythroid 2-related factor 2 (NRF2) plays a pivotal role in regulating ROS transcription, modulating the expression of numerous antioxidant genes. KEAP1 acts as a sensor for ROS levels within cells, adjusting NRF2 activity accordingly (Figure 2). Aging cells are particularly vulnerable to oxeiptosis due to their diminished antioxidant capacity. To investigate the impact of ROS accumulation on aging cells, we analyzed genes associated with oxeiptosis. Our results revealed impaired ROS elimination mechanisms in aging cells. Following ILIB treatment, a significant increase in NRF2 (Figure 2A, 0.041 ± 0.046 vs. 0.20 ± 0.19) expression was observed, suggesting an augmentation of antioxidant responses. However, there were no significant alterations in the expression levels of KEAP1 and PGAM5 (Figure 2B,C, 0.029 ± 0.03 vs. 0.022 ± 0.031; 0.073 ± 0.06 vs. 0.056 ± 0.06). Remarkably, the downstream effector AIFM1, critical for regulating oxeiptosis, exhibited a substantial decrease in expression post-ILIB treatment (Figure 2D, 0.044 ± 0.035 vs. 0.017 ± 0.01). This indicates a notable reduction in the occurrence of oxeiptosis following ILIB intervention, implying a potential therapeutic benefit in mitigating ROS-mediated cell death processes in aging cells.

### 3.3. ILIB Altered the Energy Metabolism Reprogramming of Human Cumulus Cells

**Figure 3:**
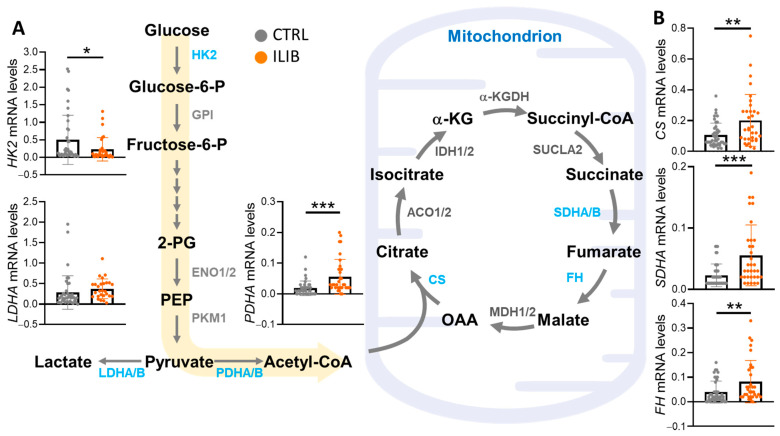
ILIB regulated the reprogramming of energy metabolism in human cumulus cells. Evaluation of the expression of ILIB altering metabolic pathways and gene levels involved in glycolysis (**A**) and the TCA cycle (**B**) by qPCR analysis. * *p* < 0.05, ** *p* < 0.01 and *** *p* < 0.001.

In this study, alterations in energy metabolism reprogramming of human cumulus cells were assessed as a result of ILIB treatment. Specifically, the expression levels of genes associated with glycolysis, such as hexokinase 2 (HK2), lactate dehydrogenase A (LDHA), and pyruvate dehydrogenase E1 subunit alpha (PDHA), as well as genes related to the TCA cycle, including CS, SDHA, and FH, were analyzed. Notably, the ILIB group exhibited a significant decrease in HK2 expression (0.22 ± 0.33 vs. 0.49 ± 0.70), while no notable difference was observed in LDHA expression, a key gene involved in lactate production (0.37 ± 0.24 vs. 0.28 ± 0.41). Conversely, PDHA expression, critical for pyruvate conversion into acetyl-Coenzyme A for aerobic respiration, showed a marked increase in the ILIB group (0.050 ± 0.053 vs. 0.019 ± 0.02) (Figure 3A). Similarly, CS levels, responsible for catalyzing citrate synthesis, exhibited a significant rise following ILIB treatment (0.20 ± 0.16 vs. 0.10 ± 0.07), as did SDHA (0.055 ± 0.049 vs. 0.021 ± 0.018) and FH (0.082 ± 0.086 vs. 0.039 ± 0.044) (Figure 3B). These results indicate that ILIB enhanced mitochondrial energy transport efficiency and boosted energy production in aging cells, highlighting its potential therapeutic benefits in mitigating age-related declines in cellular function.

### 3.4. Demographic and Clinical Characteristics of Patients before and after ILIB Treatment

**Figure 4:**
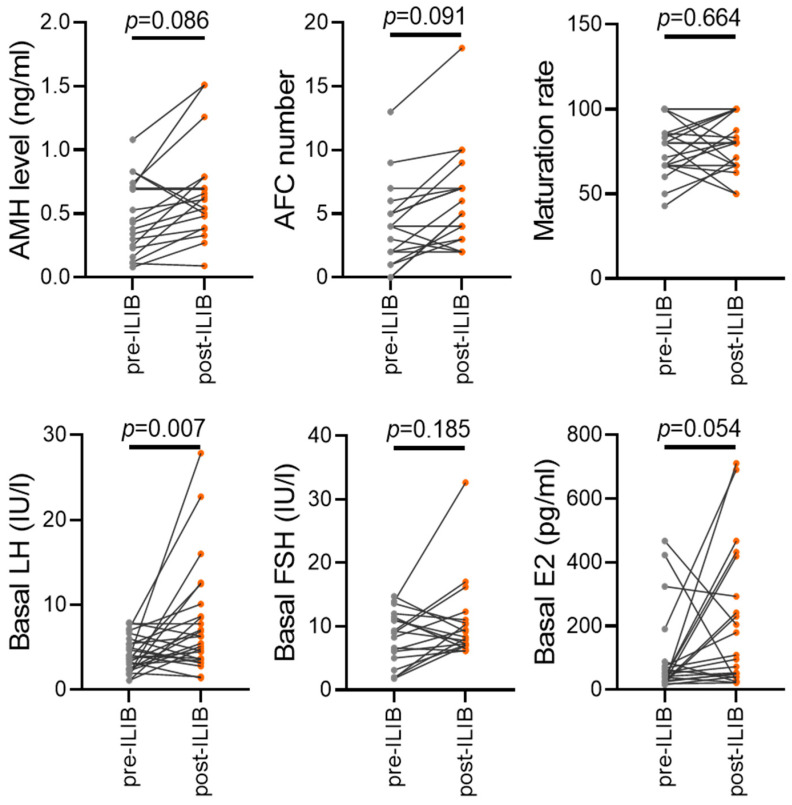
Patient clinical characteristics before and after ILIB treatment, including levels of AMH, AFC count, maturation rate, and concentrations of LH, FSH, and E2.

We meticulously examined the clinical data of patients both before and after receiving ILIB treatment, allowing for a comprehensive assessment of its effects. While the differences in AMH levels and antral follicle count (AFC) before and after ILIB treatment did not reach statistical significance, we observed a notable trend indicating an increase in these parameters post-treatment (Figure 4). This suggests a potential positive impact of ILIB on ovarian reserve and follicular development, although further investigation is warranted to elucidate these effects fully. Moreover, although there was no statistically significant difference in the maturation rate of oocytes between the pre- and post-treatment groups, an intriguing observation emerged regarding basal luteinizing hormone (LH) levels. We noted a significant increase in basal LH levels following ILIB treatment, indicating a potential modulation of hormonal profiles in response to the therapy. Furthermore, while basal follicle-stimulating hormone (FSH) and estradiol (E2) levels exhibited an upward trend after ILIB treatment, statistical significance was not achieved. Nevertheless, this trend suggests a potential influence of ILIB on hormone regulation within the ovarian environment. These findings collectively suggest that ILIB treatment may have a positive impact on ovarian function and hormonal profiles in aging patients, warranting further investigation to elucidate its mechanisms and optimize its clinical application in improving fertility outcomes.

## 4. Discussion

Emerging research has shed light on the intricate relationship between energy metabolism and ovarian aging, elucidating key molecular pathways underlying this physiological process [5]. Mitochondrial dysfunction, characterized by impaired oxidative phosphorylation and compromised ATP production, has emerged as a hallmark feature of aging ovaries [23]. The dysregulated expression of genes involved in glycolysis, the tricarboxylic acid (TCA) cycle, and oxidative phosphorylation pathways further exacerbate mitochondrial dysfunction, leading to the increased production of reactive oxygen species (ROS) and oxidative stress [24]. These aberrant metabolic processes contribute to the decline in oocyte quality and ovarian reserve observed with advancing age [25].

ILIB has gained recognition, not only for its efficacy in addressing vascular-related diseases, but also for its potential therapeutic advantages in a wide range of health conditions extending beyond cardiovascular issues [21]. Research has extensively explored its ability to improve microcirculation, enhance tissue oxygenation, and alleviate oxidative stress, particularly in conditions such as atherosclerosis and hypertension [26]. ILIB involves the exposure of blood to low-level laser light, hypothesized to trigger various physiological responses within the body. In addition to its established benefits in vascular health, ILIB has been investigated for its impact on oxidative stress, inflammation, immune function, and energy metabolism [20]. Furthermore, ILIB has emerged as a promising adjunctive therapy for chronic ailments like diabetes mellitus and autoimmune disorders. Studies indicate that ILIB may enhance glucose metabolism, alleviate diabetic neuropathy symptoms, and improve insulin sensitivity in diabetic patients [27]. Moreover, its immunomodulatory properties have shown potential in reducing disease severity and enhancing the quality of life in individuals with autoimmune conditions such as rheumatoid arthritis and systemic lupus erythematosus [28].

Previous investigations have solidified the understanding that intravascular laser blood irradiation (ILIB) possesses the capability to activate the mitochondrial respiratory chain, thus augmenting the production of adenosine triphosphate (ATP) vital for sustaining energy metabolism within aging tissues [29]. This phenomenon underscores the intricate interplay between ILIB therapy and cellular bioenergetics, suggesting a potential mechanism through which ILIB may exert its therapeutic effects. By enhancing ATP synthesis, ILIB holds promise for revitalizing cellular functions in aging tissues, potentially ameliorating age-related declines in metabolic activity and tissue function [27]. This highlights the importance of further exploring the molecular mechanisms underlying ILIB-induced metabolic enhancements and its implications for combating age-related degenerative processes.

In our investigation, we enrolled 75 infertile patients with aging ovaries and divided them into ILIB-treated and non-treated groups. The ILIB-treated group underwent two courses of laser treatment, while clinical parameters were evaluated alongside genetic analysis of cumulus cells to assess changes in oxeiptosis, glycolysis, and the TCA cycle. Our findings revealed intriguing alterations in gene expression patterns following ILIB treatment. Notably, ILIB led to a significant upregulation of oxeiptosis-related genes AIFM1 and NRF2, suggesting a potential protective effect against oxidative stress-induced cell death in aging ovaries. Furthermore, ILIB treatment resulted in a downregulation of glycolysis-associated gene HK2, indicating a shift away from anaerobic metabolism, along with increased PDHA levels, indicative of enhanced mitochondrial function. Consistent with these changes, ILIB-treated patients exhibited elevated expression of the key TCA cycle genes CS, SDHA, and FH, signifying improved energy metabolism.

The potential of ILIB to positively impact energy metabolism and cellular health in aging germ cells is a notable finding, suggesting that this therapy could be integrated into treatment protocols for improving reproductive outcomes in older individuals. The ability of ILIB to modulate oxidative stress pathways, enhance mitochondrial efficiency, and shift metabolic processes towards more efficient energy production underscores its therapeutic promise. However, it is critical to understand the underlying mechanisms in greater detail, which could pave the way for optimizing treatment protocols and maximizing therapeutic benefits. One significant limitation of our research lies in the relatively small sample size utilized. Consequently, while we observed a trend towards improvement in clinical outcomes following ILIB treatment, these changes did not reach statistical significance. As a result, it is imperative to approach the interpretation of our findings with caution and recognize the need for further investigation with larger cohorts to corroborate and validate our preliminary observations. Future studies should aim to include a more substantial number of participants and consider longitudinal designs to better assess the long-term benefits and potential side effects of ILIB therapy in the context of aging and infertility.

Moreover, exploring the differential impacts of ILIB on various subpopulations, including those with different etiologies of infertility, could provide more personalized insights and enhance the applicability of ILIB in clinical settings. Understanding patient-specific responses and tailoring treatments accordingly could significantly improve therapeutic outcomes and patient satisfaction.

## 5. Conclusions

Overall, the diverse advantages of ILIB underscore its potential as a versatile treatment approach with broader applications extending beyond ovarian aging. By addressing oxidative stress and boosting energy metabolism, ILIB holds promise for enhancing health outcomes across a range of medical conditions. This multifaceted therapy has the capacity to positively impact various health conditions by targeting underlying physiological mechanisms.

## Figures and Tables

**Table 1:** Basic characteristics of patients in the CTRL and ILIB groups.

Parameters	CTRL (*n* = 42)	ILIB (*n* = 33)
Age (years)	38.9 ± 3.2	39.8 ± 3.4
BMI (kg/m^2^)	23.3 ± 4.5	23.1 ± 4.3
Duration of infertility (years)	4.2 ± 3.3	4.9 ± 3.2
Previous IVF failure (*n*)	1.4 ± 2.3	1.8 ± 1.2
Types of infertility *n* (%)		
Primary infertility	20/42 (47.6%)	15/33 (45.5%)
Secondary infertility	23/43 (53.4%)	18/33 (54.5%)
Basal FSH (IU/L)	9.8 ± 7.5	10.9 ± 6.7
Basal E2 (pg/mL)	112.8 ± 148.8	266.5 ± 394.6
Basal LH (IU/L)	8.4 ± 13.6	7.6 ± 6.0

IVF, in vitro fertilization; FSH, follicle stimulation hormone; E2; estradiol; LH, luteinizing hormone.

**Table 2:** Cycle characteristics outcome in both groups.

Parameters	CTRL (*n* = 42)	ILIB (*n* = 33)
Stimulation duration (days)	10.6 ± 2.3	10.3 ± 2.8
No. of oocytes retrieved (*n*)	5.6 ± 4.2	4.7 ± 3.9
No. of metaphase II oocytes (*n*)	4.3 ± 4.2	3.8 ± 3.1
Maturation rate (%)	80.7 ± 3.6	84.7 ± 3.8
No. of fertilized oocytes (*n*)	3.6 ± 3.5	3.0 ± 2.7
Fertilization rate (%)	84.3 ± 3.7	78.8 ± 79.6
No. of Day 3 embryos (*n*)	3.3 ± 2.8	2.9 ± 2.7
No. of top-quality D3 embryos (*n*)	3.3 ± 2.8	2.9 ± 2.7

## Data Availability

Data are contained within the article.
